# The Unique *hmuY* Gene Sequence as a Specific Marker of *Porphyromonas gingivalis*

**DOI:** 10.1371/journal.pone.0067719

**Published:** 2013-07-02

**Authors:** Anna Gmiterek, Halina Wójtowicz, Paweł Mackiewicz, Małgorzata Radwan-Oczko, Małgorzata Kantorowicz, Maria Chomyszyn-Gajewska, Magdalena Frąszczak, Marcin Bielecki, Mariusz Olczak, Teresa Olczak

**Affiliations:** 1 Laboratory of Biochemistry, Faculty of Biotechnology, University of Wrocław, Wrocław, Poland; 2 Department of Genomics, Faculty of Biotechnology, University of Wrocław, Wrocław, Poland; 3 Department of Periodontology, Unit of Oral Pathology, Wrocław Medical University, Wrocław, Poland; 4 Department of Periodontology and Oral Medicine, Jagiellonian University, Collegium Medicum, Kraków, Poland; 5 Institute of Genetics, University of Environmental and Life Sciences, Wrocław, Poland; St. Petersburg Pasteur Institute, Russian Federation

## Abstract

*Porphyromonas gingivalis*, a major etiological agent of chronic periodontitis, acquires heme from host hemoproteins using the HmuY hemophore. The aim of this study was to develop a specific *P. gingivalis* marker based on a *hmuY* gene sequence. Subgingival samples were collected from 66 patients with chronic periodontitis and 40 healthy subjects and the entire *hmuY* gene was analyzed in positive samples. Phylogenetic analyses demonstrated that both the amino acid sequence of the HmuY protein and the nucleotide sequence of the *hmuY* gene are unique among *P. gingivalis* strains/isolates and show low identity to sequences found in other species (below 50 and 56%, respectively). In agreement with these findings, a set of *hmuY* gene-based primers and standard/real-time PCR with SYBR Green chemistry allowed us to specifically detect *P. gingivalis* in patients with chronic periodontitis (77.3%) and healthy subjects (20%), the latter possessing lower number of *P. gingivalis* cells and total bacterial cells. Isolates from healthy subjects possess the *hmuY* gene-based nucleotide sequence pattern occurring in W83/W50/A7436 (n = 4), 381/ATCC 33277 (n = 3) or TDC60 (n = 1) strains, whereas those from patients typically have TDC60 (n = 21), W83/W50/A7436 (n = 17) and 381/ATCC 33277 (n = 13) strains. We observed a significant correlation between periodontal index of risk of infectiousness (PIRI) and the presence/absence of *P. gingivalis* (regardless of the *hmuY* gene-based sequence pattern of the isolate identified [r = 0.43; *P* = 0.0002] and considering particular isolate pattern [r = 0.38; *P* = 0.0012]). In conclusion, we demonstrated that the *hmuY* gene sequence or its fragments may be used as one of the molecular markers of *P. gingivalis*.

## Introduction

Chronic periodontitis is one of the most common bacterial infections of humans, characterized by destruction of tooth-supporting tissues [1]–[3]. From the clinical point of view, periodontitis is characterized by deep periodontal pockets, resulting from the loss of connective tissue attachment and alveolar bone. The severity of the bleeding upon probing depends on the intensity of the gingival inflammation. Several studies have demonstrated that about 700 species are capable of colonizing the adult oral cavity [4]–[8]. In the subgingival plaque alone up to 400 species have been found that fall into nine bacterial phyla [9]. Analysis of bacterial species isolated from subgingival samples revealed the presence and relative abundance of periodontal pathogens, including the “red complex” bacteria (*Porphyromonas gingivalis*, *Tannerella forsythia* and *Treponema denticola*), associated with the clinical features of chronic periodontitis [5], [10]–[16]. Among several bacteria, *P. gingivalis* is considered the main etiological agent and a key pathogen responsible for initiation and progression of chronic periodontitis [17], [18]. *P. gingivalis* can enter gingival epithelial and immune cells and remain viable and capable of spreading between host cells [19]–[22], thus contributing to its survival in the oral cavity. The bacterium is a constituent of multispecies biofilm within the gingival crevice or the periodontal pocket, which also contributes to bacterial persistence and increases the probability of periodontal tissue destruction [23], [24]. *P. gingivalis* is a heme auxotroph, and therefore the uptake of this compound is essential for bacterial survival and ability to establish an infection. The bacterium acquires heme from the host environment using a novel *hmu* system comprising the HmuY hemophore and five additional proteins [25]–[28].

Given the emerging evidence of an association between periodontal infections and systemic conditions such as diabetes mellitus, rheumatoid arthritis, cardiovascular and respiratory diseases, and pre-term low birth weight [29]–[34], detection of *P. gingivalis* may be also important for assessment of the above-mentioned conditions. It has been demonstrated that periodontal pathogens, including *P. gingivalis*, not only may colonize oral cells, but also have been identified in atherosclerotic plaques in coronary arteries [35], [36].

Several different methods have been used for qualitative and quantitative analysis of oral pathogens, including in situ hybridization, DNA hybridization, and real-time quantitative PCR (qPCR). Among them, real-time PCR is not only sensitive and reliable but also a fast, efficient, and inexpensive method for detection of pathogens in human specimens [6], [10], [37]–[39]. To detect bacteria, usually the 16S ribosomal RNA gene (*16S rRNA*) has been used, because nucleotide sequences of these genes are well conserved among many bacterial species [40], [41]. However, using this target, it is impossible to distinguish between highly related species. For this reason, several other bacterial DNA sequences have been used to design more specific primers, such as the DNA-dependent RNA polymerase gene (*rpoB*) [42], [43] or the fimbrillin A gene (*fimA*) [44].

To detect *P. gingivalis* in polymicrobial human samples, specific, accurate and sensitive methods are still required, since identification of this bacterium is important not only for the study of chronic periodontitis, but also for severe systemic conditions. In addition, in polymicrobial infections bacteria are subjected to different antibiotic treatments and therefore it is demanded to develop sensitive methods that classify these bacteria to particular species. For this purpose, we first performed extensive phylogenetic analysis of *P. gingivalis* HmuY protein and *hmuY* gene. Then, we developed a simple but efficient assay for specific and sensitive detection of *P. gingivalis* using the *hmuY* gene sequence. Based on our results it seems that the unique *hmuY* sequence may serve as one of the molecular markers of *P. gingivalis*.

## Materials and Methods

### Patients and Collection of Samples

The use of human subjects and study protocol were approved by the Ethical Committee of Wroclaw Medical University (451/2009) and the Jagiellonian University Bioethical Committee in Krakow (KBET/157/B/2009). All subjects were required to read and sign a written consent form. Subgingival samples were collected from 66 patients with advanced chronic periodontitis (CP), possessing at least 14 teeth (age from 32 to 79 years). The control group consisted of 40 healthy control (NP) subjects (age from 22 to 51 years). Both clinical and radiological examinations were performed. Evaluation of periodontium was carried out using assessment of the hygiene level by the approximal plaque index (API %) [45], and the level of inflammation by the sulcus bleeding index (SBI %) [45]. Diagnosis of periodontitis was based on the recommendations of standards for periodontal disease definition [46]. The pocket depth probing and furcation involvement were evaluated in order to assess the severity of periodontitis. Then, taking into consideration these two scores, the periodontal index of risk of infectiousness (PIRI) was estimated [47]. Samples from chronic periodontitis patients were collected from active sites (periodontal pocket depth ≥5 mm at interproximal sites and bleeding upon probing). Exclusion criteria of the study were: healthy periodontium, less than 14 teeth (allowing the best diagnosis of periodontitis and evaluation of PIRI), smoking, pregnancy and nursing, presence of systemic disease, general antibiotic treatment and periodontal treatment (scaling and/or curettage) within two months before the investigation. Samples from healthy subjects with no periodontal pockets and no evidence of inflammation and attachment loss at any sites were also collected as a control. In the group comprising patients with chronic periodontitis (CP), subgingival samples were collected with a sterile curette from the deepest periodontal pockets and in the control group (NP) from molar/premolar interdental spaces. All samples were placed in Eppendorf tubes containing sterile phosphate buffered saline (PBS) and stored at –20°C until further analysis.

### Bacterial Strains and Growth Conditions

*P. gingivalis* W83 was cultured anaerobically in Schaedler broth (SB, Biocorp, Poland) as described previously [48]. *Bacteroides fragilis* NCTC 9343 was maintained in Brain Heart Infusion (BHI, Biocorp) broth medium supplemented with 0.5% yeast extract and 15 µg/ml hemin at 37°C under anaerobic conditions (10% H_2_, 5% CO_2_ and 85% N_2_). *Prevotella intermedia* 17 and *Tannerella forsythia* ATCC 43037 were grown in Triptic Soy Broth (TSB, Biocorp) with 0.5% yeast extract, 0.05% cysteine, 0.5 mg/ml hemin and 2 µg/ml vitamin K at 37°C under anaerobic conditions.

### Database Searches for HmuY Homologs and Phylogenetic Analyses

To obtain the essential knowledge about diversity and evolution of *P. gingivalis hmuY* and its homologs, which is necessary to develop PCR-based detection of this species based on this gene, we carried out extensive phylogenetic analysis. To gather all potential homologs to *P. gingivalis* HmuY available in sequence databases, comprehensive searches of GenBank in different ways were carried out. The homologs were collected according to online Psi-Blast searches with *E-value*<0.005 and local searches of Conserved Domain Database [49] for the HmuY domain with every sequence present in the non-redundant protein database. All sequences annotated as ‘HmuY’ were also extracted from GenBank. After elimination of redundant sequences from the joint set, 258 sequences showing significant similarity to the HmuY domain with *E-value*≤0.005 were chosen. Incomplete or fragmentary sequences were excluded from further analyses. Finally, 103 amino acid sequences representing all phyletic lineages to infer the global phylogenetic trees were selected. In addition, we reconstructed detailed phylogenetic trees based on amino acid and nucleotide sequences from all available closest homologs to *P. gingivalis* HmuY, including new sequences isolated in this study from human subjects. The amino acid alignments were obtained in MAFFT 6.925b using slow and accurate algorithm L-INS-i with 1,000 cycles of iterative refinement [50] and were edited manually in JalView [51]. Nucleotide sequences of the closest homologs to *P. gingivalis* HmuY were aligned according to their amino acid alignment.

Phylogenetic trees based on amino acid sequences were inferred by the Bayesian approach in PhyloBayes 3.3e [52], as well as the maximum likelihood method used in TreeFinder [53] and morePhyML 1.14 using PhyML 3.0 [54], [55]. In PhyML, we applied the LG+Γ(5) model of amino acid substitutions in the global tree reconstruction and the LG+F+Γ(5) model in inferring the tree for the closest HmuY homologs. The models were selected according to ProtTest 3.2 [56] assuming optimization of models, branches, and topology of the tree. In the TreeFinder approach, we applied the MIX+F+Γ(5) model in the global tree reconstruction and the LG+F+Γ(5) model for the tree of the closest HmuY homologs. The models were chosen according to the Propose Model module in this program assuming optimized frequencies of amino acids. In PhyloBayes analyses, we assumed the LG+Γ(5) model for the two tree reconstructions. Two independent Markov chains were run through 200,000 cycles in inferring the global tree and 100,000 cycles in the reconstruction of the tree for the closest HmuY homologs. After obtaining convergence, the last 100,000 and 50,000 trees from each chain, respectively, were collected to compute a posterior consensus.

To infer phylogenetic trees based on nucleotide alignments six approaches using five programs – MrBayes 3.2.1 [57], PhyloBayes 3.3e [52], TreeFinder [53], morePhyML 1.14 based on PhyML 3.0 [54], [55] and PAUP* 4.0b [58] – were applied. In MrBayes analyses, we assumed three separate mixed+I+Γ(5) models for three codon positions to sample appropriate models across the substitution model space in the Bayesian MCMC analysis itself [59], avoiding the need for a priori model testing. In this analysis, two independent runs starting from random trees, each using 4 Markov chains, were applied. Trees were sampled every 100 generations for 10,000,000 generations. In the final analysis, trees from the last 5,007,000 generations that reached the stationary phase and convergence (*i.e.* the standard deviation of split frequencies stabilized and lower than 0.005, much below the proposed threshold of 0.01) were selected. In the PhyloBayes approach, two independent Markov chains were run for 500,000 cycles assuming the CAT Poisson+Γ(5) model with number of components, weights and profiles inferred from the data. After obtaining a convergence, the last 200,000 trees from each chain were collected to compute the posterior consensus. In TreeFinder tree reconstruction, we applied separate substitution models for three codon positions – HKY+Γ(5) (for the first codon position), HKY (for the second codon position), and HKY+Γ(5) (for the third codon position) – as suggested by this program’s Propose Model module. The tree inferred with (more)PhyML as well as maximum likelihood and neighbor joining trees calculated in PAUP were based on the best-fit substitution model 012034+F+Γ(5) as found in jModeltest 2.1 [60] among 1624 candidate models.

For both amino acid and nucleotide alignments, search depth set to 2 in TreeFinder and the best heuristic search algorithms, NNI and SPR, in (more)PhyML were applied. In the maximum likelihood method in PAUP, the final tree was searched from 10 starting trees obtained by stepwise and random sequence addition followed by the tree-bisection-reconnection (TBR) branch-swapping algorithm. Edge support was estimated by the bootstrap analysis with 1,000 replicates in each of these three programs.

### DNA Extraction, Standard Polymerase Chain Reaction (PCR) Amplification and DNA Sequencing

Total genomic DNA was extracted directly from human samples or bacterial cultures using the NucleoSpin Tissue kit (Macherey-Nagel, Germany) according to the instructions for hard-to-lyse bacteria. DNA concentration and purity were determined spectrophotometrically using a UV-visible spectrophotometer (NanoPhotometer Pearl; Implen, Germany).

Nucleotide sequences of the *hmuY* gene sequences of *P. gingivalis* strains (381, ATCC 33277, W50, W83, A7436, TDC60) and their flanking regions were obtained from the database of the National Center for Biotechnology Information (NCBI). To sequence the entire *hmuY* gene extracted from isolates present in the human oral cavity, primers (all primers used in this study were synthesized by Genomed, Poland) for standard PCR were designed for the flanking or internal regions of the gene (Table 1). DNA products were amplified using genomic DNA and standard PCR (Biometra, Germany) under the following conditions: initial denaturation at 94°C for 5 min and 35 cycles of denaturation at 94°C for 30 s, primer annealing at 64°C for 30 s, extension at 72°C for 1 min, and final extension at 72°C for 5 min. The reaction mixture contained 2×PCR Master MixPlus (A&A Biotechnology, Poland), 40 pmol of each reverse and forward primer and 5 µl of template (final volume 50 µl). Aliquots of 45 µl of PCR reaction mixture were separated on 1.5% agarose gels in the presence of ethidium bromide in TAE buffer (4 mM Tris-acetate, 1 mM EDTA) at 100 V for 25 min. Amplification products were isolated using the Gel Out kit (A&A Biotechnology) and sequenced (Genomed).

**Table 1:** Primers used in this study.

Primer	5′→3′ nucleotide sequence	Description	Source
HYq4-F	GCTTCGAAATACGAAACGTG	Forward primer used for standard and quantitative PCR to amplify fragment of*P. gingivalis hmuY* gene	This study
HYq4-R	TATATCCGTCTGTCGGAACG	Reverse primer used for standard and quantitative PCR to amplify fragment of*P. gingivalis hmuY* gene	This study
fHYb2–F	ACCATAAACACACGGAATAATCG	Forward primer used for standard PCR to amplify the entire *P. gingivalis hmuY* gene	This study
HYAGf2–R	GATATTGCCGGATACGATGG	Reverse primer used for standard PCR to amplify the entire *P. gingivalis hmuY* gene	This study
16SrRNA–F	CTTGACTTCAGTGGCGGCAG	Forward primer used for standard and quantitative PCR to amplify the*P. gingivalis*-specific region of the *16S rRNA* gene	[61]
16SrRNA–R	AGGGAAGACGGTTTTCACCA	Reverse primer used for standard and quantitative PCR to amplify the *P. gingivalis*-specific region of the *16S rRNA* gene	[61]
16Suni-F	GTGsTGCAyGGyTGTCGTCA	Forward *16S rRNA* gene-based universal primer used for standard and qPCR to amplifyfragment of bacterial *16S rRNA* genes	[61]
16Suni-R	ACGTCrTCCmCACCTTCCTC	Reverse *16S rRNA* gene-based universal primer used for standard and qPCR to amplifyfragment of bacterial *16S rRNA* genes	[61]
16SrRNA-F1	CATCGGTAGTTGCTAACAGTTTTCG	Forward primer used for standard PCR to amplify the *P. gingivalis*-specific region of the *16S rRNA* gene	[39]
16SrRNA-R1	CCGACCTCTACATTATCAG	Reverse primer used for standard PCR to amplify the *P. gingivalis*-specific region of the*16S rRNA* gene	[39]
16Suni-F1	GGATTAGATACCCTGGTAGTC	Forward *16S rRNA* gene-based universal primer used for standard PCR to amplify fragment of bacterial *16S rRNA* genes	[39]
16Suni-R1	TACCTTGTTACGACTT	Reverse *16S rRNA* gene-based universal primer used for standard PCR to amplifyfragment of bacterial *16S rRNA* genes	[39]

**Figure 1:**
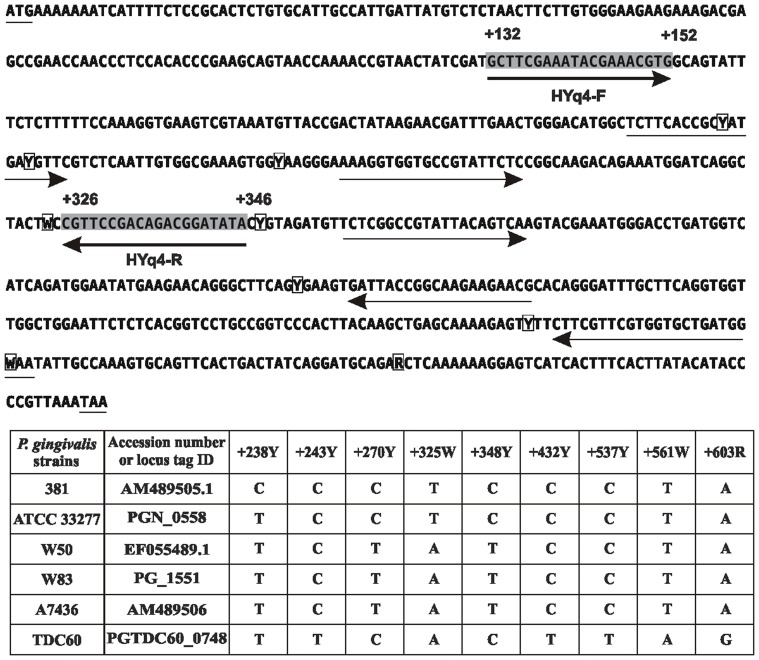
Comparison of nucleotide sequences of the *hmuY* gene identified in database-deposited sequences. Sequences in gray boxes with thick arrows below show a set of *hmuY* gene-based primers (HYq4-F and HYq4-R) used to detect the desired target. Thin arrows show other sequences of primers checked at the preliminary stage, but not used in the final experiments because of cross reactivity with other targets. Accession numbers and DNA sequence variations of the *hmuY* gene (Y and W) are listed in the table.

A set of primers to exclusively amplify the *P. gingivalis hmuY* fragments was designed based on the nucleotide sequences of the gene (Table 1 and Fig. 1). Specificity of the primers was examined by standard PCR using genomic DNA or bacterial cultures as a template. To allow efficient DNA denaturation, bacterial cells were first added to water and heated at 100°C for 1 min, and then all reagents were added. PCR was carried out under the following conditions: initial cycle of denaturation at 94°C for 5 min and 35 cycles of denaturation at 95°C for 30 s, primer annealing at 64°C for 30 s, extension at 72°C for 1 min, and final extension at 72°C for 5 min. Aliquots of 15 µl of PCR reaction mixture were separated on 1.5% agarose gel in the presence of ethidium bromide and visualized using the GelDoc system (Bio-Rad, USA). As a reference, a routinely used set of *P. gingivalis 16S rRNA* gene-based primers (regions corresponding to +648 and +667 or +1006 and +1025 nucleotide positions) was employed (Table 1) with PCR conditions described by Maeda *et al.* [61]. Amplification products were separated in 1.5% agarose gels, isolated and sequenced (Genomed).

### Real-time PCR

Real-time PCR was performed using a Stratagene Mx3005P thermocycler (Agilent Technologies, USA). Amplification was carried out in triplicate in a 15-µl reaction mixture comprising 5 µl of DNA extracted from bacterial cultures or human samples, 20 pmol of each primer and 2×Brilliant SYBR Green QPCR Master Mix (Agilent Technologies). Amplification was carried out under the following conditions: initial denaturation at 94°C for 10 min and 40 cycles of denaturation at 95°C for 20 s, primer annealing at 64°C for 15 s, extension at 72°C for 20 s. Amplification plots were recorded and the specificity of the reaction was determined using denaturation curve analysis in the temperature range 65–94°C. Real-time PCR amplification with *P. gingivalis 16S rRNA* gene-based primers (Table 1) was carried out as described by Maeda *et al.* [61]. The sensitivity test of primers was performed using a standard curve generated by qPCR of 5-fold dilutions of genomic DNA extracted from *P. gingivalis* W83 strain under conditions described above. The total number of bacterial cells was quantified using a universal primer set (Table 1) designed based on bacterial *16S rRNA* genes under conditions described by Maeda *et al.* [61]. Number of *P. gingivalis* or total bacterial cells was determined in 5 µl of originally extracted genomic DNA samples and calculated from the standard curve generated using genomic DNA extracted from *P. gingivalis* W83 strain.

### Statistical Analysis

Clinical parameters of examined subjects and the number of bacterial cells in human samples were analyzed using Mann-Whitney-Wilcoxon test. Pearson’s correlation coefficients were calculated and to assess whether there was a relationship between the parameters examined correlation tests were used and the significance level α = 0.005 was used. In addition, to assess the relationship between considered variables linear regression was applied. To find the model best fitting the data, the Akaike information criterion (AIC) was used. The best model was as follows: Y = 0.25779+0.01202× X1+0.28933× X2, where Y denotes the index of isolate equal to 1, 2, 3, and 0 for 381/ATCC 33277, W50/W83/A7436, TDC60 (strains are listed according to their increasing virulence), and *P. gingivalis* not detected, respectively, X1 represents SBI and X2 denotes PIRI. To test the significance of a particular regression coefficient the significance level α = 0.005 was used. Statistical analysis was performed using the statistical package R (R: A Language and Environment for Statistical Computing, R Core Team, www.r-project.org). The data obtained from qPCR analysis were analyzed using Stratagene MxPro software (Agilent Technologies).

## Results and Discussion

### Patients

Clinical characteristics of all subjects included in this examination are shown in Table 2. In the group comprising patients with chronic periodontitis (CP) the oral hygiene was mainly poor. The API value was in the range 14–22% only in 7 patients, and in others ranged from 26% to 100%. Bleeding index was lower than 15% in only 10 patients. In other patients this value was from 17% to 100% and the PIRI index from 1 to 8. In the periodontitis group, 17% of patients were classified in the group of high risk of infectiousness (PIRI from 6 to 10). In the control group (NP), the API index value was from 0% to 70%, SBI index from 0% to 31%, and the PIRI index value was 0 (except one subject with a value of 2 related to the presence of 2 furcations). Statistically significant differences were found between patients and healthy subjects regarding API, SBI and PIRI (*P*<0.001).

**Table 2:** Clinical characteristics of patients with chronic periodontitis and healthy control subjects.

Group	Number (male/female)	Age (years)	API^a^ (%)	SBI^b^ (%)	PIRI^c^
Patients with chronic periodontitis	66 (19/47)	50.31±9.24	54.72±27.94^d^	48.45±32.15^d^	4.55±1.53^d^
Control healthy subjects	40 (20/20)	24.92±6.87	18.65±20.69	5.49±7.91	0.05±0.32

a API, Approximal Plaque Index.

b SBI, Sulcus Bleeding Index.

c PIRI, Periodontal Index of Risk of Infectiousness.

d *P*<0.001 (patients *versus* healthy subjects). Data are shown as mean±standard deviation (mean±SD).

### Phylogenetic Analyses and Sequence Variation of *HmuY*

The aim of this study was to develop a *hmuY* gene-based real-time PCR assay employing SYBR Green chemistry for detection of *P. gingivalis* in human polymicrobial samples. To fulfill this aim, knowledge about diversity and evolution of nucleotide and amino acid sequences of *P. gingivalis* HmuY and its homologs in other bacteria, especially those present in the oral cavity, is required. Therefore, we first thoroughly gathered all significant homologs to *P. gingivalis* HmuY and performed global phylogenetic analyses based on the set of 103 representative protein sequences (Fig. S1). Although the vast majority of homologs to HmuY were found among Bacteroidetes, the sequences were also represented by γ- and δ-Proteobacteria as well as Spirochaetes. Single cases of HmuY sequences were detected in β-Proteobacteria, Gemmatimonadetes and related to Bacteroidetes, Ignavibacteria and Chlorobi. Interestingly, one significant hit to HmuY was reported by an uncultured euryarchaeote isolated from marine metagenome and classified in marine group II.

Although deep branches of obtained trees were generally poorly supported in bootstrap analysis because of a very high divergence of analyzed sequences, many of the same clades were recovered by trees inferred by different methods (Fig. S1). Most Bacteroidetes sequences formed one big clade and clearly separated from proteobacterial sequences occupying a basal position in the tree. Interestingly, some sequences from different taxonomic groups were clustered together, which suggests horizontal transfer of *hmuY* between bacteria. Such clusters were created by δ-Proteobacteria and Spirochaetes, Bacteroidetes and δ-proteobacterium *Sorangium cellulosum* So ce56, Spirochaetes and Bacteroidetes. Sequences from three Bacteroidetes classes, Cytophagia, Flavobacteria and Sphingobacteria, were distributed into two clades separated by sequences from Bacteroidia (and some Flavobacteria). Such tree topology indicates an ancient duplication of *hmuY* before divergence of the main Bacteroidetes lineages. Additional, more recent duplications of this gene must have also happened because many strains were represented by more than one closely related gene copy (e.g. *Capnocytophaga gingivalis* ATCC 33624 by four and *Pedobacter* sp. BAL39 by five copies). The presence of several *hmuY* copies suggests some functional differentiation of their products, which should be verified by experimental studies. The HmuY from *P. gingivalis* (together with two *P. asaccharolytica* DSM 20707 sequences) was placed at the base of other Bacteroidia sequences represented by *Bacteroides*, *Prevotella* and *Tannerella* (Fig. S1).

**Figure 2:**
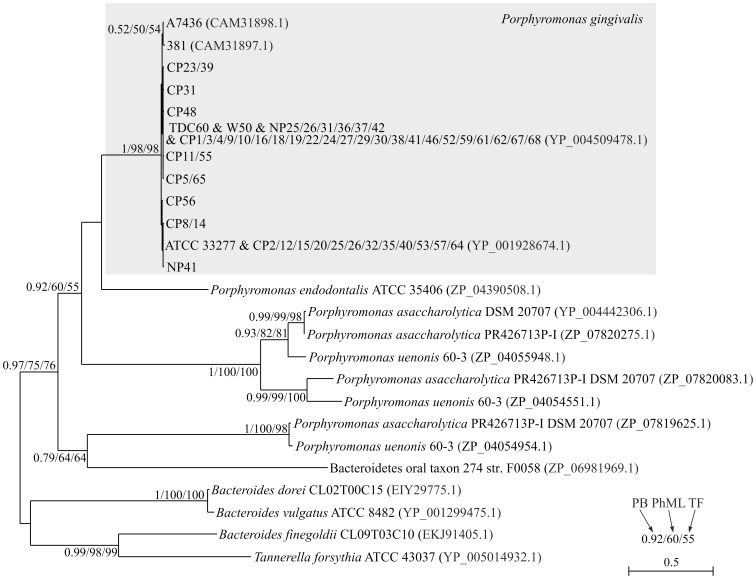
PhyloBayes tree based on amino acid alignment of closest homologs to *P.* *gingivalis* HmuY. The clade of *P. gingivalis* strains and isolates is marked by the gray rectangle. Joined names of strains and isolates indicate their 100% sequence identity. Numbers at nodes, in the order shown, correspond to posterior probabilities estimated in PhyloBayes (PB), as well as bootstrap support values calculated in PhyML (PH) and TreeFinder (TF). Values of the posterior probabilities and bootstrap percentages lower than 0.50 and 50%, respectively, were omitted or indicated by a dash “–”.

**Figure 3:**
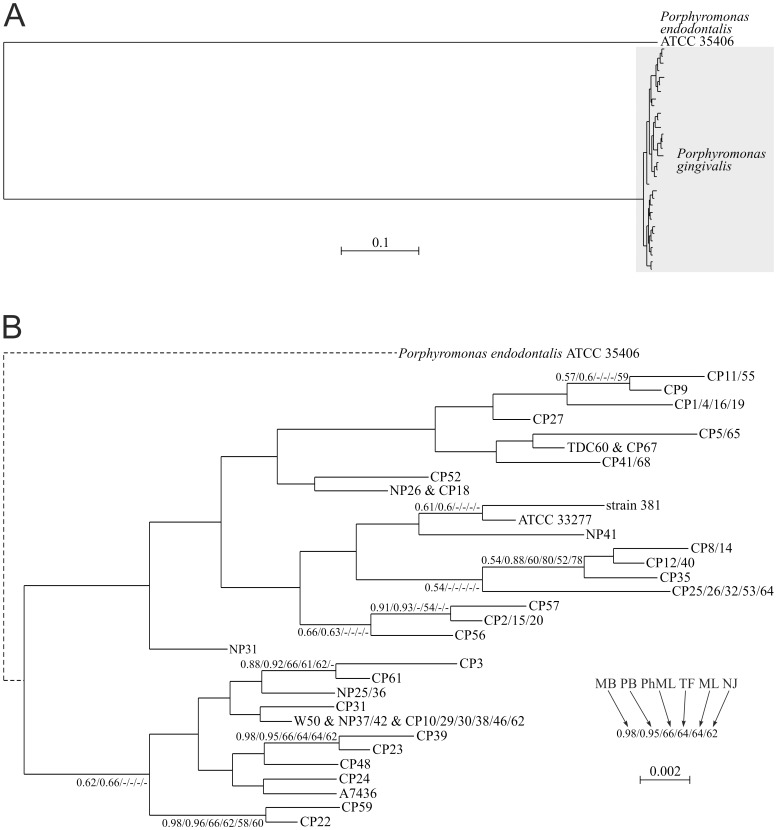
MrBayes tree based on nucleotide alignment of closest homologs to *P.* **gingivalis hmuY**** **.** **** A) The general tree with the real branch length leading to *P. endodontalis* from the common ancestor of *P. gingivalis* clade (marked by the gray rectangle). B) The tree with the branch length leading to *P. endodontalis* shortened 100 times (the dashed line) to show detailed relationships between *P. gingivalis* strains and isolates. Joined names of strains and isolates indicate their 100% sequence identity. Numbers at nodes, in the order shown, correspond to posterior probabilities estimated in MrBayes (MB) and PhyloBayes (PB), as well as bootstrap support values calculated in PhyML (PH), TreeFinder (TF) and PAUP using maximum likelihood (ML) and neighbor joining (NJ) methods. Values of the posterior probabilities and bootstrap percentages lower than 0.50 and 50%, respectively, were omitted or indicated by a dash “–”.

To study in detail phylogenetic relationships between *P. gingivalis* HmuY and its closest homologs, we carried out additional analyses based both on amino acid (Fig. 2) and nucleotide (Fig. 3) alignments, including new sequences isolated from patients examined in this study. For this purpose, the entire *hmuY* gene was amplified using genomic DNA isolated from human samples. Subsequently, known nucleotide sequences of the *hmuY* deposited in databases were compared with data obtained from sequencing analysis. All *Porphyromonas* sequences clearly separated from other Bacteroidia sequences with high and moderate support (Fig. 2). One sequence with unspecified taxonomic affiliation described as Bacteroidetes oral taxon 274 str. F0058 (ZP_06981969.1) was grouped with *Porphyromonas* sequences, which suggests that it may represent a hitherto unknown *Porphyromonas* taxon. Interestingly, *P. asaccharolytica* DSM 20707 and *P. uenonis* 60-3 were represented by three HmuY homologs, which were distributed in three significantly supported clades. Each of these clades included one sequence from these two species, which indicates that two gene duplications must have happened before divergence of these taxa. However, no additional HmuY homolog was found for *P. gingivalis* although four genomes of this species (W83, ATCC 33277, TDC60, JCVI SC001) were completely sequenced. All analyzed *P. gingivalis* sequences created one significant monophyletic group showing very little divergence at the amino acid level. Many of the studied sequences were identical and differed in four sites at most (min. identity was 98.1%). Among 58 analyzed sequences only 12 were unique. In contrast to that, identity between sequences of *P. gingivalis* and other *Porphyromonas* species was much smaller and equaled 38.0% on average (min.: 33.8%, max.: 49.8%). This indicates a very high evolutionary rate of HmuY between *P. gingivalis* and other *Porphyromonas* species but very low among *P. gingivalis* strains or isolates.

This conclusion is more pronounced at the nucleotide level. Standard Blastn searches of GenBank for homologs to *P. gingivalis hmuY* did not find any nucleotide sequences from other *Porphyromonas* species, even if the smallest word size = 7 was assumed to increase sensitivity. Phylogenetic analysis of these sequences revealed a very long distance (almost 1.7 nucleotide substitutions per site on average) between the clear clade of *P. gingivalis hmuY* sequences and their closest homolog from *P. endodontalis* (Fig. 3). In the obtained trees, sequences from *P. gingivalis* appeared to be a very homogeneous and poorly diversified group. Minimum identity between these sequences was only 98.1% and the maximum difference was only 12 positions (Fig. S2, Table S1). Among 58 analyzed sequences only 32 were unique. For comparison, the *P. gingivalis* sequences differed from the *P. endodontalis* homolog in 269 positions on average (min.: 265, max.: 274). The average identity to these sequences was 55.8% (min.: 55.0%, max.: 56.5%).

These results indicate that the potential origin of the amplified samples of *hmuY* sequences from species other than *P. gingivalis* is highly improbable because the sequences obtained are almost identical to other sequences classified independently by other authors also to *P. gingivalis*. Some of these sequences come from completely sequenced strains that must have been pure and well characterized cultures. Moreover, the high sequence identity of the *hmuY* gene between *P. gingivalis* strains or isolates in comparison to the low sequence similarity to its homologs found in other bacteria demonstrates that invariant fragments of the DNA sequence encoding this gene can be successfully used to design primers to specifically detect *P. gingivalis* in polymicrobial human samples. Therefore, a set of primers (HYq4-F and HYq4-R) was designed to match the regions which are highly conserved among database-deposited *hmuY* gene sequences (Fig. S2), but different from HmuY homologs encoded in other bacterial species (Figs. 2, 3, and Fig. S1). In fact, the sequence of the reverse primer dedicated to the *P. gingivalis hmuY* gene differs from the closest *P. endodontalis* homolog in seven positions whereas it is identical in all analyzed *P. gingivalis* strains and isolates (Fig. S2). In the case of the forward primer, there are six different positions in the *P. endodontalis* homolog in comparison to the *P. gingivalis* consensus and only two unique *P. gingivalis* sequence isolates (*i.e.* CP31 and NP25/NP36) showed one transition G→A (Fig. S2). The specificity of the designed primers can be confirmed by Blastn searches of sequence databases using the primers as a query. Although the searches found bacterial sequences with 100% identity in the length of 18 nt (*Spirosoma linguale*) or 17 nt (*Streptococcus agalactiae*, *Naumovozyma castellii*, *Serratia*) and with 95% identity along the full length of 20 nt (*Azospirillum lipoferum*, *Bacteroides salanitronis*), they showed similarity only to one primer per taxon. Each of two primers showed 100% identity on the length of 16 nt to the complete genome of *Hahella chejuensis*. However, the found sequences were separated by almost 250 kb and could not amplify even this long region because they had outward (instead of inward) orientation to each other and this region.

We also performed *in silico* analyses of the designed primers using mFOLD [62] assuming folding (*i.e.* annealing) temperature 64°C. The obtained results indicated that the formation of potential secondary structures is highly unfavorable in these primers. The Gibbs free energy change (ΔG) was 0.26 kcal/mol, −0.82 kcal/mol, 0.91 kcal/mol and –0.98 kcal/mol for primers HYq4-F (internal hairpin), HYq4-R (internal hairpin), fHYb2–F (3′-end hairpin) and HYAGf2–R (internal hairpin), respectively. These values are much larger than the assumed thresholds for SYBR Green primer design, i.e. −3 kcal/mol and –5 kcal/mol for 3′-end and internal hairpin structures, respectively. Therefore, the influence of these structures on primer sensitivity is negligible.

### Experimental Analysis of Specificity and Sensitivity of Designed Primers

**Figure 4:**
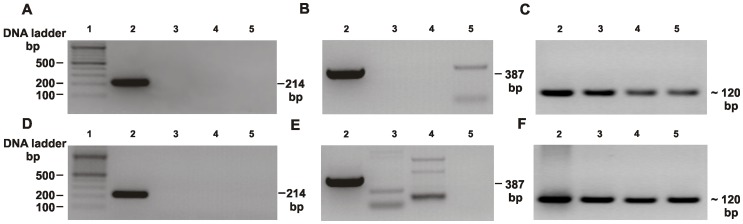
Specificity of the *hmuY*-based primers analyzed by standard PCR. DNA fragments were amplified using genomic DNA (A, B, and C) or bacterial cultures (D, E, and F) and separated in agarose gels. Lanes: 1) 100 bp DNA marker, 2) *P. gingivalis* W83, 3) *T. forsythia* ATCC 43037, 4) *P. intermedia* 17, 5) *B. fragilis* NCTC 9343. Amplification products obtained using *hmuY* gene-based primers (A and D), *P. gingivalis 16S rRNA* gene-based primers (B and E), and universal *16S RNA* gene-based primers (C and F) [61].

Standard PCR and real-time PCR are now widely used to detect pathogens in human infections; therefore in this study we designed a set of primers for specific detection of *P. gingivalis*. As controls, we used selected strains belonging to *Bacteroides, Prevotella* and *Tannerella*, possessing the closest HmuY homologs, namely *B. fragilis* and two periodontopathogens, *P. intermedia* and *T. forsythia*. The specificity of the HYq4-F and HYq4-R primers was first tested using standard PCR and genomic DNA extracted from *P. gingivalis* W83 strain and other bacterial species examined. The data showed that the distinct PCR product at the expected size (214 bp) was amplified only in the case of *P. gingivalis* (Figs. 4A and 4D). To confirm the presence of *P. gingivalis*, similar experiments were performed, but using *P. gingivalis 16S rRNA* gene-based primers selected upon literature search [6], [39], [41], [63]–[65] and our preliminary experiments (data not shown). In final experiments, we used primers designed by Maeda *et al.* [61] because of the following reasons: 1) sequences of primers were derived from the conserved internal region of *P. gingivalis 16S rRNA* gene, 2) primers were designed to amplify short DNA sequences suitable for real-time PCR analysis, 3) primers allowed us to obtain amplification products at highest quality compared to other primers tested, 4) variability of respective points in standard curves using DNA extracted from *P. gingivalis* only, as well as in standard curve using DNA extracted from *P. gingivalis* and contaminating strains was very low compared to other primer sets, 5) samples were collected in the same way as in our study. Results from experiments carried out with primers designed by Maeda *et al.* [61] showed amplification of the desired target at the expected size (387 bp) in the case of *P. gingivalis* (Figs. 4B and 4F). However, some unspecific amplification products were obtained using DNA extracted from other species or whole bacterial cultures of these species.

**Figure 5:**
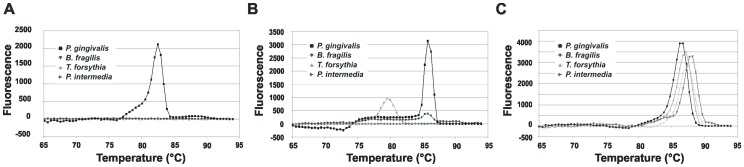
Specificity of *P.* *gingivalis hmuY* gene-based primers analyzed by quantitative real-time PCR (qPCR). DNA fragments were amplified using genomic DNA and *P. gingivalis hmuY* gene-based primers (A), *P. gingivalis 16S rRNA* gene-based primers (B), and universal *16S RNA* gene-based primers (C) [61].

The specificity test of *hmuY* gene-based primers was also performed using real-time PCR with SYBR Green chemistry. The denaturation curve analysis showed that unspecific PCR products were not amplified since a sharp peak at the expected T_m_ (82.87±0.23) was observed only when genomic DNA of *P. gingivalis* was used as a template (Fig. 5A). Analogous experiments carried out with primers specific for the *16S rRNA* gene of *P. gingivalis* [61] resulted in a specific product amplified when *P. gingivalis* was examined (T_m_ = 85.77°C). However, as shown in Fig. 5B, a peak at the same T_m_ (85.30°C), but with lower intensity, was observed for *B. fragilis*. In the case of *P. intermedia*, a peak at lower T_m_ (79.55°C) was visible. Our data indicated that *16S rRNA* gene-based primers might be less specific for detection of *P. gingivalis*, as compared to *hmuY* gene-based primers.

The sensitivity test of *P. gingivalis hmuY* gene-based primers was carried out using qPCR with 5-fold dilutions of genomic DNA extracted from *P. gingivalis* W83 strain generating the standard curve (data not shown). The *hmuY* gene-based primer set designed in this study detected as low as 0.41 fg of *P. gingivalis* genomic DNA in 5-µl samples and the amplification was linear in the range from 4 ng to 10.2 fg. Assuming that the genome size of *P. gingivalis* W83 is 2.34 Mb, the primer set detected 10.2 fg of genomic *P. gingivalis* DNA and amplification plots showed a minimal limit of 3.99 cells from *P. gingivalis* W83 in 5 µl of DNA samples. Using the TaqMan probe method and primers designed for the conservative lipopolysaccharide-encoding gene, Hyvarinen *et al.* [66] demonstrated that the detection limit of qPCR primers was 40 fg of genomic *P. gingivalis* DNA. Lyons *et al.* [39] reported the quantitative range of *P. gingivalis* as 10^2^–10^8^ cells using nested PCR with a TaqMan probe and *16S rRNA* gene-based primers. Although the TaqMan method is thought to be more specific compared with the SYBR Green method, several studies have demonstrated that the latter could be reasonably used in studies on periodontal pathogens. Park *et al.* [43] detected 4 fg of genomic *P. gingivalis* DNA using primers designed for the *rpoB* gene and a dye-based qPCR. Using both TaqMan and SYBR Green methods with *16S rRNA* gene-based primers, Sakamoto *et al.* [6] demonstrated quantitative detection of five periodontal bacteria in the range of 10^3^–10^8^ cells, and Maeda *et al.* [61] detected three periodontal species over a range of 10–10^7^ cells, while the quantitative range for total bacteria was 10^2^–10^7^ cells. Taking all together, our data demonstrated that the primer set based on the *hmuY* gene sequence is highly specific and sensitive for *P. gingivalis* detection.

### Detection of *P. gingivalis* in Human Samples

**Figure 6:**
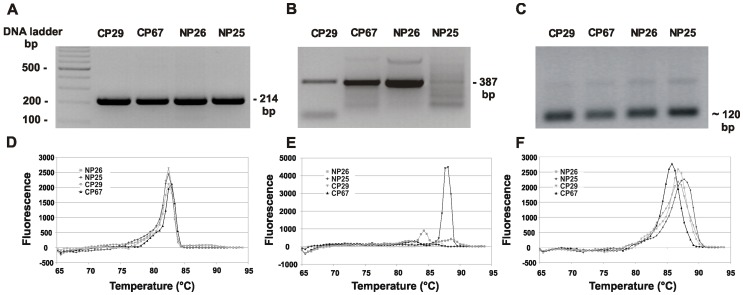
Detection of *P.* *gingivalis* in human samples. Standard PCR (A, B, and C) and qPCR (D, E, and F) with genomic DNA as a template were used to amplify desired targets. Amplification performed with *P. gingivalis hmuY* gene-based primers (A and D), *P. gingivalis 16S rRNA* gene-based primers (C and E), and universal *16S RNA* gene-based primers (D and F) [61]. NP23 and NP26 samples from representative control healthy subjects possessing *P. gingivalis*; CP43 and CP66, samples from representative patients with chronic periodontitis possessing *P. gingivalis*.

To detect *P. gingivalis* in human samples, first standard PCR with the set of *hmuY* gene-based primers was used. As expected, *P. gingivalis* was identified in the majority of samples collected from chronic periodontitis patients (CP, 77.3%, n = 51), compared to control subjects who did not have periodontal disease (NP, 20%, n = 8) (Figs. 6A and 6D, and data not shown). These results were confirmed by standard PCR with *P. gingivalis 16S rRNA* gene-based primers designed by Maeda *et al.* [61]. However, the latter set of primers was less specific since in the addition of amplification of the target DNA sequence, also some unspecific products were produced (Figs. 6B and 6E, and data not shown).

Accumulation of unspecific amplification products using *P. gingivalis 16S rRNA* gene-based primers may result in invalid assessment of the number of *P. gingivalis* cells and lead to false positive results. Therefore, to demonstrate putative application of *hmuY* gene-based primers in determination of *P. gingivalis* cells in human samples qPCR was used. For this purpose, amplification results from 3 serial dilutions of DNA extracted from human subgingival samples in the linear range of the assay were averaged and compared with the standard curve generated during the same experiment. The correlation between *P. gingivalis* cell number and periodontal health status has been reported by several investigators, demonstrating that advanced stage of periodontitis may be related with higher number of this bacterium. Our intent was to verify such results, check how general is this rule, and if it is also valid in the cases studied by us using newly designed set of primers. Our results showed that, compared with healthy subjects possessing *P. gingivalis* (2.12×10^4^±1.74×10^4^, range 2.37×10^5^–6.74×10^7^), patients with chronic periodontitis possess a higher number of this bacterium (1.61×10^6^±2.49×10^6^, range 2.70×10^6^–2.16×10^9^). We also determined total bacterial cell number using universal primers designed by Maeda *et al.* [61]. Universal set of primers allowed PCR amplification when DNA extracted from *P. gingivalis* or from other bacteria examined, as well as whole bacteria, and DNA extracted from human samples were used as a template (Figs. 4C, 4F, 5C, 6C, and 6F). Total number of oral bacterial cells estimated using qPCR and universal primers was higher in samples derived from patients (9.93×10^9^±1.03×10^10^, range 2.14×10^8^–3.08×10^10^), compared with samples derived from control subjects (7.88×10^9^±9.63×10^9^, range 5.37×10^7^–3.04×10^10^). Similar to published data [5], [10]–[14], the number of *P. gingivalis* cells, as well as total number of bacterial cells determined in each sample varied by a few orders of magnitude. Therefore, these data should be viewed with caution and the following reasons should be taken into consideration. Determination of absolute total bacterial cells by qPCR could be influenced by the variation in the number of *16S rRNA* gene copies in a given species, since it has been shown that it may vary from 1 to 15 [67]–[72], and in this study we assumed the presence of 4 copies in all bacteria. Thus, total bacterial counts evaluated by the number of 16S rDNA may be fluctuated in polymicrobial samples. Quantification by qPCR also requires knowledge of the size of the genome and since this information is unknown for many oral bacteria, we assumed that the genome size of other bacteria was similar to *P. gingivalis*. In addition, variations in the number of bacteria are caused by different sampling techniques used and levels of bacteria analyzed in subgingival samples are average values, usually much higher at sites of disease activity. In our study, such effect may be also caused by comparison of control group including only 8 *P. gingivalis*-positive samples with group of patients consisting of as many as 51 positive samples. All these issues point to the importance of development of internal standards allowing truly quantitative studies.

Epidemiologic studies demonstrated that *P. gingivalis* strains vary in their virulence, with strains being more virulent (e.g. W83, W50, and A7436, the latter highly similar to W83) or less virulent (e.g. 381, ATCC 33277) [73]–[75]. TDC60 strain has been isolated from a severe periodontal lesion of a patient and exhibited higher pathogenicity in causing abscesses in mice than strains W83 and ATCC 33277 [76]. In addition, it has been shown that there is a correlation between *P. gingivalis* strains and tissue destruction level with *P. gingivalis* W83 strain contributing to periodontitis to a greater extent than other strains [77]–[79]. Therefore in this study we employed *hmuY* gene analysis to demonstrate potential differences in the nucleotide sequences between *P. gingivalis* isolates and ascribe them to particular known *P. gingivalis* strains. For this purpose the most profound differences in the nucleotide sequence of the *hmuY* gene (Fig. 1 and Fig. S2) were taken into consideration. We found that in the group comprising healthy subjects the most prevalent isolates possessed differences in the *hmuY* nucleotide sequence, being mainly a combination of those occurring in W83/W50/A7436 strains (n = 4) or 381/ATCC 33277 strains (n = 3), compared with the pattern typical for TDC60 strain (n = 1) (Figs. 1, 2, and Fig. S3). In the group of patients with chronic periodontitis the most prevalent isolates possessed the nucleotide *hmuY* sequence pattern typical of TDC60 strain (n = 21) or a combination of W83/W50/A7436 strains (n = 17). In patients carrying *P. gingivalis* the pattern typical for 381/ATCC 33277 strains was less common (n = 13). Based on these results, it can be assumed that TDC60 or W83/W50/A7436 are potentially more virulent strains, which is reflected in the *hmuY* nucleotide sequence pattern. Based on our results we also found a correlation between PIRI and the presence of a particular isolate (TDC60, W83/W50/A7436, 381/ATCC 33277 strains; listed according to their increasing virulence), or the absence of *P. gingivalis* (r = 0.38; *P* = 0.0012). Regardless of the identified isolate, the correlation coefficient between the presence/absence of *P. gingivalis* and PIRI equaled 0.43 (*P* = 0.0002). All our data demonstrated that the *hmuY* gene-based identification of the presence of *P. gingivalis* may be correlated with an advanced stage of periodontitis.

### Conclusions

In this study we have presented extensive phylogenetic analysis of *P. gingivalis* HmuY protein and the *hmuY* gene. Based on phylogenetic analyses and examination of human specimens, we demonstrated the potential use of the set of *hmuY* gene-based primers and real-time PCR with SYBR Green for detection of *P. gingivalis*. We propose that the *hmuY* gene region may serve as one of the molecular markers of *P. gingivalis*. However, it should be noted that adjunctive methods, for example a combination of *P. gingivalis* markers, may be required to unequivocally identify *P. gingivalis* in polymicrobial human specimens. The assay described in this study does not have the capability for accurate quantitative measurements of bacterial cell numbers in polymicrobial samples. Precise quantitative PCR-based methods would require proper internal standards, so far not developed.

## Supporting Information

Figure S1 **PhyloBayes tree based on amino acid alignment of HmuY homologs.** Representatives of main bacterial groups are marked in different colors and *P. gingivalis* was additionally bolded. Numbers at nodes, in the order shown, correspond to posterior probabilities estimated in PhyloBayes (PB), as well as bootstrap support values calculated in PhyML (PH) and TreeFinder (TF). Values of the posterior probabilities and bootstrap percentages lower than 0.50 and 50%, respectively, were omitted or indicated by a dash “–”.
(PDF)

Figure S2 **Alignment of closest homologs to** ***P. gingivalis hmuY*** **.** Joined names of strains and isolates indicate their 100% sequence identity. Arrows above the alignment indicate position of designed primers.
(PDF)

Figure S3 **Comparison of** ***P. gingivalis*** **HmuY identified in database-deposited sequences and in isolates from human subjects.** Differences in nucleotide (A) or amino acid sequences (B) are shown in black boxes. A) Y = T or C; K = T or G; W = T or A; B = T or G or C; B) X = an amino acid as defined by the respective codon. Pg strains, *P. gingivalis* strains (A7436, W50, W83, TDC60, ATCC 33277, 381); Samples, *P. gingivalis* isolates present in human samples.
(PDF)

Table S1 **Percentage identity between nucleotide sequences of closest homologs to** ***P. gingivalis hmuY*** **.** Joined names of strains and isolates indicate their 100% sequence identity.
(XLS)
