# Supplementary figures and images for: The Unique hmuY Gene Sequence as a Specific Marker of Porphyromonas gingivalis
Source: PLoS One. 2013 Jul 2;8(7):e67719. doi: 10.1371/journal.pone.0067719 (PMC3699645; doi:10.1371/journal.pone.0067719)

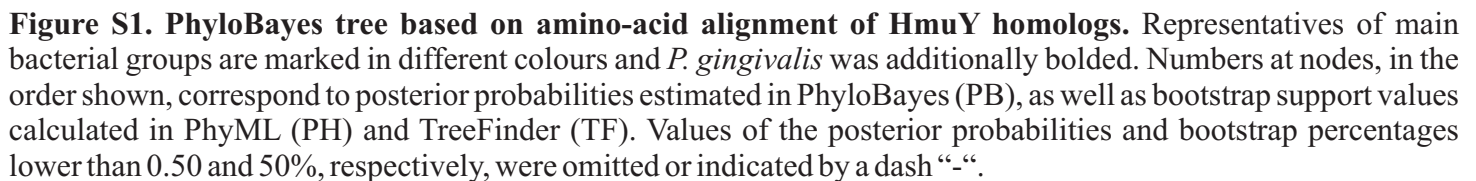

Supplement: Figure S1 — PhyloBayes tree based on amino acid alignment of HmuY homologs. Representatives of main bacterial groups are marked in different colors and P. gingivalis was additionally bolded. Numbers at nodes, in the order shown, correspond to posterior probabilities estimated in PhyloBayes (PB), as well as bootstrap support values calculated in PhyML (PH) and TreeFinder (TF). Values of the posterior probabilities and bootstrap percentages lower than 0.50 and 50%, respectively, were omitted or indicated by a dash “–”. (PDF) [file pone.0067719.s001.pdf]
