# Supplementary material for: The Unique hmuY Gene Sequence as a Specific Marker of Porphyromonas gingivalis
Source: PLoS One. 2013 Jul 2;8(7):e67719. doi: 10.1371/journal.pone.0067719 (PMC3699645; doi:10.1371/journal.pone.0067719)
Supplement: Figure S2 — Alignment of closest homologs to P. gingivalis hmuY . Joined names of strains and isolates indicate their 100% sequence identity. Arrows above the alignment indicate position of designed primers. (PDF) [file pone.0067719.s002.pdf]

Figure S2. Alignment of closest homologs to *P. gingivalis hmuY*. Joined names of strains and isolates indicate their 100% sequence identity. Arrows above the alignment indicate position of designed primers.

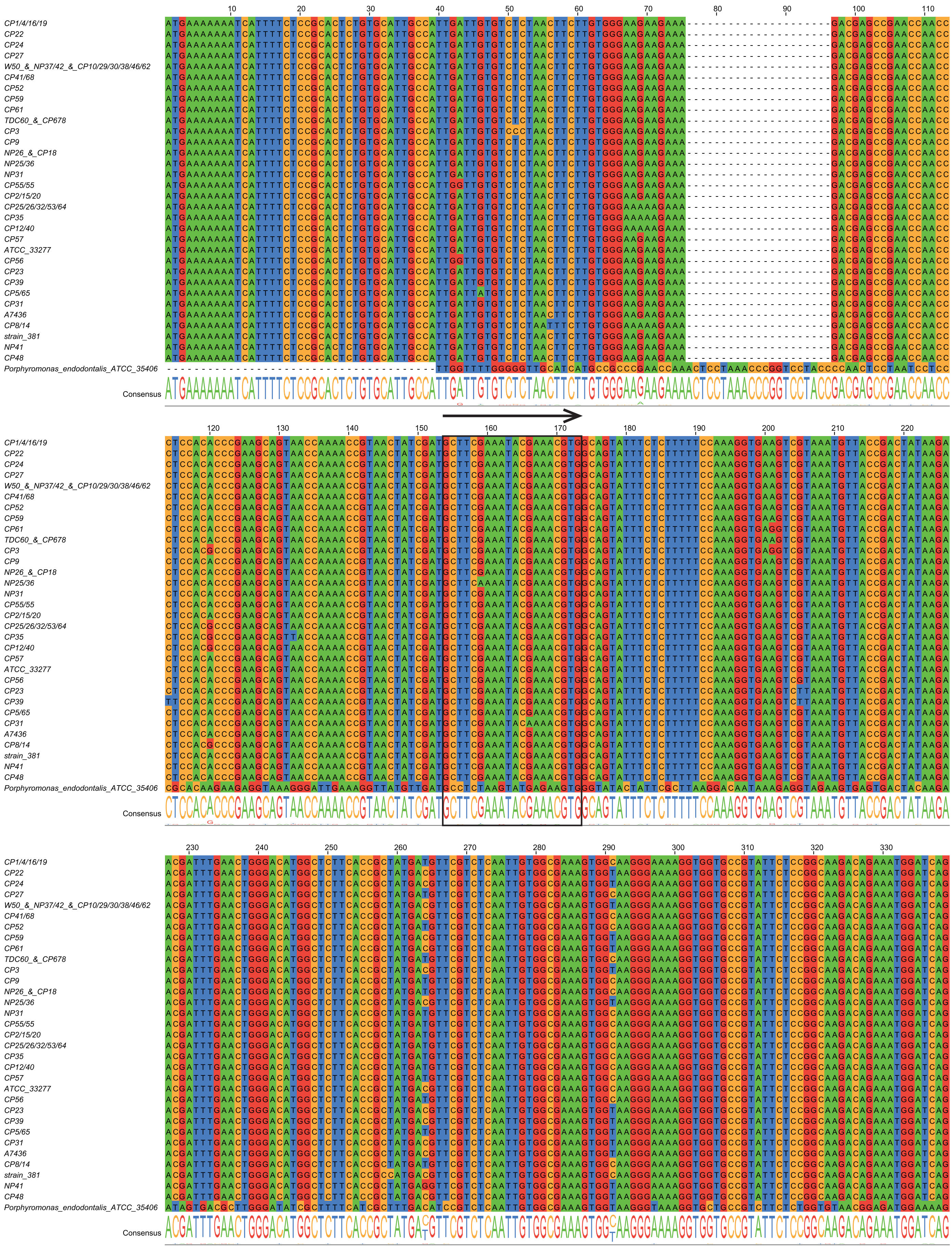

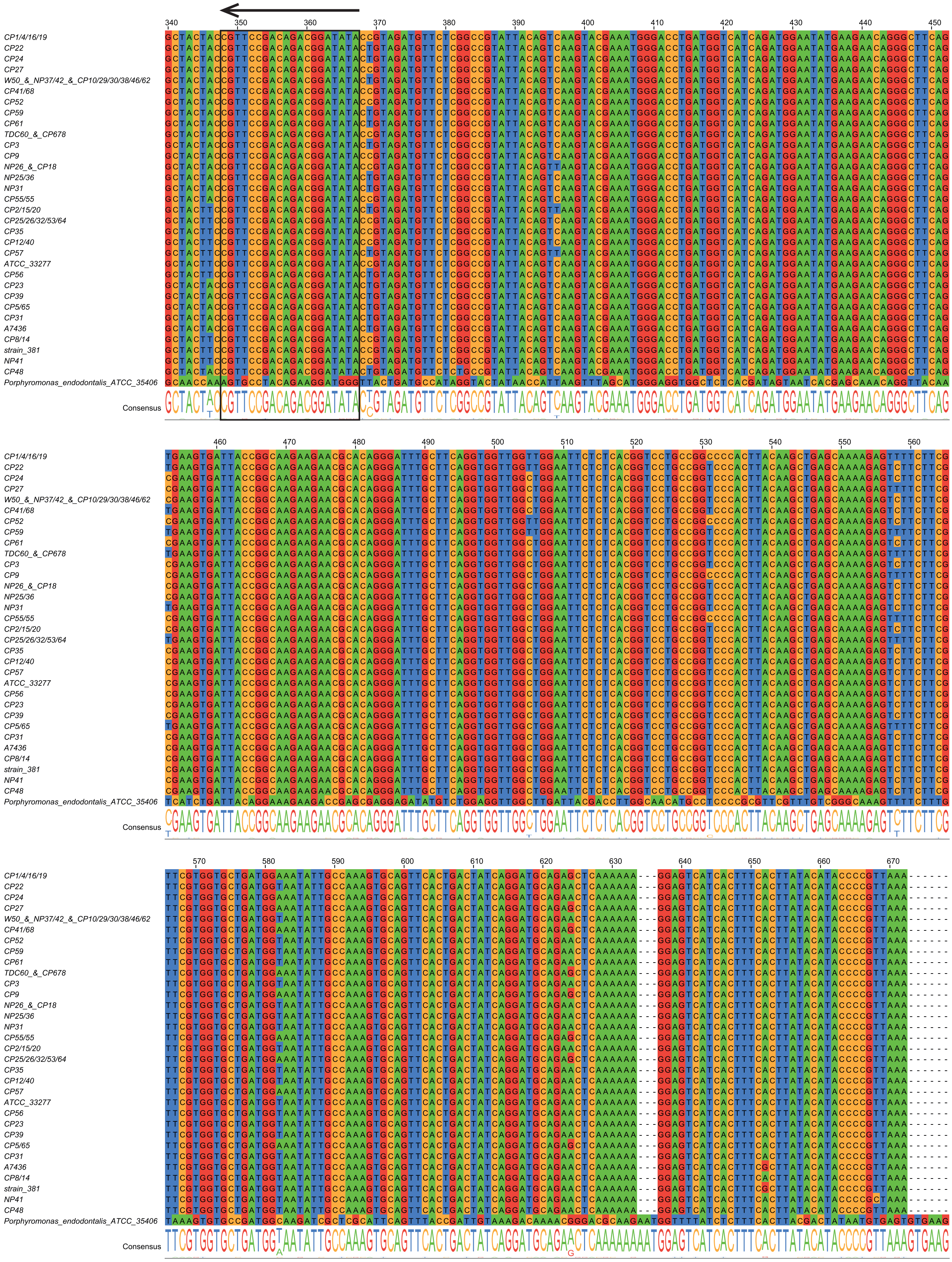
340
350
360
370
380
390
400
410
420
430
440
450
CP1/4/16/19
CP22
CP24
CP27
W50_&_NP37/42_&_CP10/29/30/38/46/62
CP41/68
CP52
CP59
CP61
TDC60_&_CP678
CP3
CP9
NP26_&_CP18
NP25/36
NP31
CP55/55
CP2/15/20
CP25/26/32/53/64
CP35
CP12/40
CP57
ATCC_33277
CP56
CP23
CP39
CP5/65
CP31
A7436
CP8/14
strain_381
NP41
CP48
Porphyromonas_endodontalis_ATCC_35406
Consensus
460
470
480
490
500
510
520
530
540
550
560
570
580
590
600
610
620
630
640
650
660
670
