# Supplementary material for: The Unique hmuY Gene Sequence as a Specific Marker of Porphyromonas gingivalis
Source: PLoS One. 2013 Jul 2;8(7):e67719. doi: 10.1371/journal.pone.0067719 (PMC3699645; doi:10.1371/journal.pone.0067719)
Supplement: Figure S3 — Comparison of P. gingivalis HmuY identified in database-deposited sequences and in isolates from human subjects. Differences in nucleotide (A) or amino acid sequences (B) are shown in black boxes. A) Y = T or C; K = T or G; W = T or A; B = T or G or C; B) X = an amino acid as defined by the respective codon. Pg strains, P. gingivalis strains (A7436, W50, W83, TDC60, ATCC 33277, 381); Samples, P. gingivalis isolates present in human samples. (PDF) [file pone.0067719.s003.pdf]

A

```
Pg strains  A T G A A A A A A A T C A T T T T C T C C G C A C T C T G T G C A T T G C C A T T G A T T G T G T C T C T A A C T T C T T G T G G G A A G A
samples     A T G A A A A A A A T C A T T T T C T C C G C A C T C T G T G C A T T G C C A T T G A T T A T G T C Y C T A A Y T T C T T G T G G G A A G A

Pg strains  A G A A A G A C G A G C C G A A C C A A C C C T C C A C A C C C G A A G C A G T A A C C A A A A C C G T A A C T A T C G A T G C T T C G A A
samples     A G A A A G A C G A G C C G A A C C A A C C Y T C C A C A C C C G A A G C A G W A A C C A A A A C C G T A A C T A T C G A T G C T T C G A A

Pg strains  A T A C G A A A C G T G G C A G T A T T T C T C T T T T T C C A A A G G T G A A G T C G T A A A T G T T A C C G A C T A T A A G A A C G A T
samples     A T A C G A A A C G T G G C A G T A T T T C T C T T T T T C C A A A G G T G A A G T C K T A A A T G T T A C C G A C T A T A A G A A C G A T

Pg strains  T T G A A C T G G G A C A T G G C T C T T C A C C G C Y A T G A Y G T T C G T C T C A A T T G T G G C G A A A G T G G Y A A G G G A A A A G
samples     T T G A A C T G G G A C A T G G C T C T T C A C C G C T A T G A B G T T C G T C T C A A T T G T G G C G A A A G T G G Y A A G G G A A A A G

Pg strains  G T G G T G C C G T A T T C T C C G G C A A G A C A G A A A T G G A T C A G G C T A C T W C C G T T C C G A C A G A C G G A T A T A C Y G T
samples     G T G G T G C C G T A T T C T C C G G C A A G A C A G A A A T G G A T C A G G C T A C T W C C G T T C C G A C A G A C G G A T A T A C Y G T

Pg strains  A G A T G T T C T C G G C C G T A T T A C A G T C A A G T A C G A A A T G G G A C C T G A T G G T C A T C A G A T G G A A T A T G A A G A A
samples     A G A T G T T C T C K G C C G T A T T A C A G T Y A A G T A C G A A A T G G G A C C T G A T G G T C A T C A G A T G G A A T A T G A A G A A

Pg strains  C A G G G C T T C A G Y G A A G T G A T T A C C G G C A A G A A G A A C G C A C A G G G A T T T G C T T C A G G T G G T T G G C T G G A A T
samples     C A G G G C T T C A G Y G A A G T G A T T A C C G G C A A G A A G A A C G C A C A G G G A T T T G C T T C A G G T G G T T G G Y T G G A A T

Pg strains  T C T C T C A C G G T C C T G C C G G T C C C A C T T A C A A G C T G A G C A A A A G A G T Y T T C T T C G T T C G T G G T G C T G A T G G
samples     T C T C T C A C G G T C C T G C C G G Y C C C A C T T A C A A G C T G A G C A A A A G A G T Y T T C T T C G T T C G T G G T G C T G A T G G

Pg strains  W A A T A T T G C C A A A G T G C A G T T C A C T G A C T A T C A G G A T G C A G A A C T C A A A A A A G G A G T C A T C A C T T T C G C T
samples     W A A T A T T G C C A A A G T G C A G T T C A C T G A C T A T C A G G A T G C A G A G C T C A A A A A A G G A G T C A T C A C T T T C A C T

Pg strains  T A T A C A T A C C C C G T T A A A T A A
samples     T A T A C A T A C C C C G Y T A A A T A A
```

B

```
Pg strains  M K K I I F S A L C A L P L I V S L T S C G K K K D E P N Q P S T P E A V T K T V T I D A S K Y E T W Q Y F S F S K G E V V N V T D Y K N D
samples     M K K I I F S A L C A L P L I M X L X S C G K K K D E P N Q X S T P E A X T K T V T I D A S K Y E T W Q Y F S F S K G E V X N V T D Y K N D

Pg strains  L N W D M A L H R X D V R L N C G E S G K G K G G A V F S G K T E M D Q A T X V P T D G Y T V D V L G R I T V K Y E M G P D G H Q M E Y E E
samples     L N W D M A L H R Y X V R L N C G E S X K G K G G A V F S G K T E M D Q A T X V P T D G Y X V D V L X R I T X K Y E M G P D G H Q M E Y E E

Pg strains  Q G F S E V I T G K K N A Q G F A S G G W L E F S H G P A G P T Y K L S K R V F F V R G A D G N I A K V Q F T D Y Q D A E L K K G V I T F T
samples     Q G F X E V I T G K K N A Q G F A S G G W X E F S H G P A X P T Y K L S K R X F F V R G A D X N I A K V Q F T D Y Q D A E L K K G V I T F T

Pg strains  Y T Y P V K
samples     Y T Y P X K
```

Figure S3. Comparison of *P. gingivalis* HmuY identified in database-deposited sequences and in isolates from human subjects. Differences in nucleotide (a) or amino-acid sequences (b) are shown in black boxes. (a) Y = T or C; K = T or G; W = T or A; B = T or G or C; (b) X = an amino acid as defined by the respective codon. Pg strains, *P. gingivalis* strains (A7436, W50, W83, TDC60, ATCC 33277, 381); Samples, *P. gingivalis* isolates present in human samples.
